# Supplementary material for: Harmonizing corporate carbon footprints
Source: Nat Commun. 2021 Oct 22;12:6149. doi: 10.1038/s41467-021-26349-x (PMC8536701; doi:10.1038/s41467-021-26349-x)
Supplement: Supplementary file 2 — Description of Additional Supplementary Files [file 41467_2021_26349_MOESM2_ESM.docx]

Description of Additional Supplementary Files

Title: Supplementary Data 1

Description: The supplementary data sheet contains all data and calculations underlying this manuscript. It provides a detailed overview how the results are derived.
